# Annotations

**Published:** 1888-05-12

**Authors:** 


### Annotations.

**Sources of Small-pox.**

Some instructive reading is furnished by the annual report of the Ambulance Committee of the Metropolitan Asylums Board. It is not pleasant to learn that with very few exceptions the cases of small-pox which occurred during the past year were directly traceable to infected persons arriving in London from the Continent or from the provinces. It ought to be possible to prevent such cases from being a source of danger to others. It is not very comforting either to be informed that of 106 people sent to the hospitals of the Metropolitan Asylums Board as small-pox cases no fewer than 44 were sent back at once to their homes, because the disease from which they were suffering was not small-pox at all. That is to say, in nearly half the instances an error of diagnosis was made. Those 44 persons whose cases were wrongly diagnosed were obliged in consequence of this mistake to ride in an ambulance used for small-pox cases, and perhaps along with one or more people who were actually suffering from the disease at the moment. They were taken into the hospital where were patients with every kind of infectious fever. It is not improbable that some who went into the hospital quite free from any taint of small-pox went away anything but free. Perhaps it is impossible to prevent mistakes in every instance, but it is eminently desirable that they should be reduced to a minimum. It is more satisfactory to know, on the other hand, that although outbreaks of small-pox occurred in as many as seventeen of the thirty parishes and unions comprising the metropolitan district during the year 1887, they did not in any instance, thanks to the energy with which they were combated, assume serious proportions. The rule is as soon as information is obtained as to the probable cause of an outbreak, to communicate with the medical officer of the district concerned. "This has, no doubt," in the words of the report, "materially assisted in the success of the measures taken to prevent the spread of the disease." Much is done by the metropolitan authorities to deal promptly with outbreaks as they occur; and those patients who are in poor circumstances are managed with a degree of care, kindness, and skill which are beyond all praise. Notwithstanding the criticisms which are sometimes very freely uttered, the public may be congratulated on the promptitude and efficiency which dangerous fevers have been dealt with in recent years, and the comparative immunity enjoyed by the inhabitants of the metropolis generally. The difficulties of the tasks with which the Board is confronted are often enormous, and it cannot be denied that within the last few years the results have been in many cases surprisingly good.

**Calmer Weather at the Rotherham Hospital.**

There has been for some time past a considerable amount of feeling among the working-men supporters of the Rotherham Hospital, caused by the non-election of one of their number, Mr. G. De Borde, to his former place on the Committee. Since the establishment of the institution, the working men of Rotherham have contributed no less than a third of the total amount raised for its maintenance. They have, therefore, considered themselves entitled to a large measure of direct representation, and their claim has been allowed. Last year Dr. Foote, one of the honorary surgeons, who has served the institution for many years, was unable, in consequence of illness, to perform his usual duties for a time. Temporary arrangements were made to supply his lack of service, but Mr. De Borde was not content with these, and, contrary to the wishes of his fellow-managers, moved the appointment of a fourth honorary surgeon. The authorities showed their disapproval of Mr. De Borde's action by not re-electing him a member of the weekly board. The working men, on their part, resented the slight to their representative, and, as they not unnaturally supposed, to themselves. Here were the elements of a pretty little storm. A meeting of the representatives of the workmen was recently held in the board-room, and the whole question came up for consideration. The working men attended in large numbers, prepared to maintain the rights of themselves and their deputy. The Rev. Dr. Falding presided, and by his candour and tact entirely dispelled the rising suspicions of the workmen. He showed them that if Mr. De Borde had not been re-elected it was partly in consequence of his own somewhat impulsive action, and partly also by an oversight. There was, he explained, a quite sufficient honorary staff to perform all the duties of the hospital efficiently in the temporary absence of Dr. Foote. He expressed his own sympathy and respect for working men, and his conviction that the interests of the hospital were furthered by a full representation of the working class element. Speaking as a Liberal and a Nonconformist, he denied absolutely the allegation that there had been any attempt on the part of the Church and Conservative party to claim an undue share of authority in the conduct of affairs. Dr. Falding's judicious and conciliatory address put everybody into good humour, and the threatened storm ended in the finest of weather. It would be a great misfortune if anything were done at Rotherham or elsewhere to diminish the keen interest of the operative class in the prosperity of hospitals. There is a movement in every part of the country in favour of largely increasing such interest, and that movement is one of the most important departures for medical charities which recent times have witnessed.

**A Real University for London.**

No medical man's name appears among the members of the Royal Commission appointed to inquire into the question of the establishment of a teaching University for London. We have more than once given expression to what is generally understood among people who know that London has a present no University, but only a board of examiners and labellers. It would be just as proper to call a publishers' reader an author as to call the Examining Board of Burlington House a University. Common sense is beginning to prevail; and some of the most eminent men of the time in science, law, and divinity have been constituted a Royal Commission to take evidence and report. But not a single medical man's name appears on the list. What does this mean? It cannot possibly mean that it is proposed to constitute a new University in the largest city in the world without a medical faculty. Nor can it mean that no medical man has been invited to sit upon the commission. What does it mean? Is it that every physician and surgeon of eminence is determined to set up two united Medical Colleges, with degree-granting powers, and to call them by what would in that case be the absurd name of university? Is medicine again to be separated from the whole field of human culture, as it so long was at Oxford and Cambridge, to its utter and complete degradation as a science and a learned profession? English medicine for centuries was a disgrace to Englishmen, and that principally because it was shut out from a broad and liberal culture by its exclusion from the universities. Is the same mistake to be made again? How is it that so many of the most eminent of London physicians and surgeons are, in everything but their specialty of medicine or surgery, merely "general practitioners writ large?" Because their education has been almost exclusively medical. Because they do not know anything of the mental agility which comes from the study of law, of the intellectual breadth which philosophy gives to her votaries, or of the noble humanity which is the inspiration of him who has devoted time and thought to the profounder mysteries of divinity. Doctors, and particularly London doctors, are, as a rule, specialists only; eminent specialists if you will, but still specialists. They smell of their specialty at all times. They know little, and apparently care nothing for the general studies and interests of mankind. That is to their degradation as men, and to the diminution of their capacity even as specialists. Is it now really a purpose among London medical men to separate medicine finally and entirely from general culture in the future? If such be the object of the Colleges of Physicians and Surgeons, that object must be resisted by the general voice of the profession to the utmost. Medical men have for centuries been stifled and suffocated by medicine. It is time for them to be men as well as doctors. They *must be men first* if they are ever to reach the highest degree of efficiency of which their art is capable.

